# Supplementary material for: Temporal Comparative Transcriptome Analysis on Wheat Response to Acute Cd Toxicity at the Seedling Stage
Source: Plants (Basel). 2023 Feb 1;12(3):642. doi: 10.3390/plants12030642 (PMC9921683; doi:10.3390/plants12030642)

Supplementary Figure S1. Heatmap for ABA hormone-related genes.

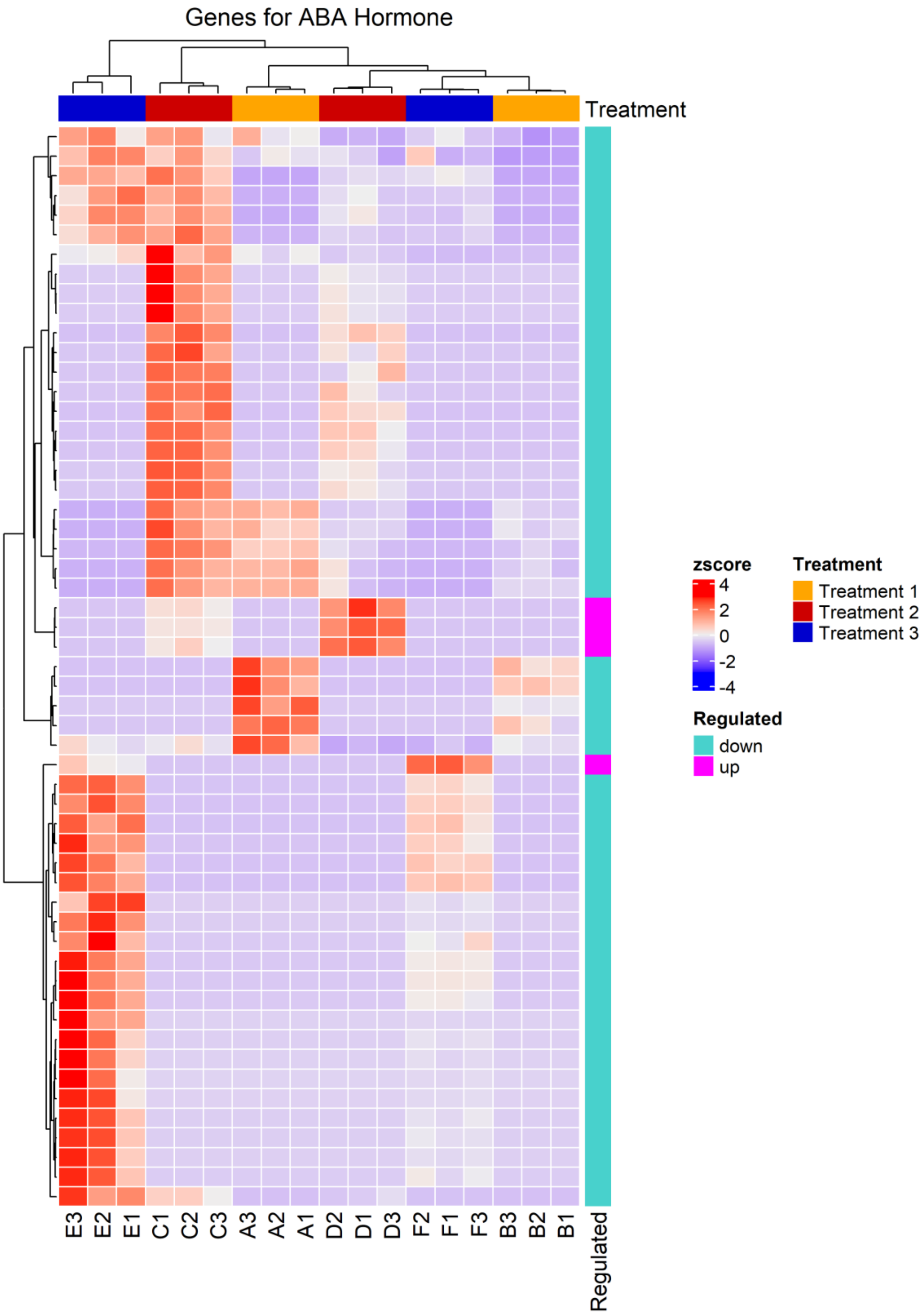

Supplementary Figure S2. Heatmap for Specific heavy metal genes.

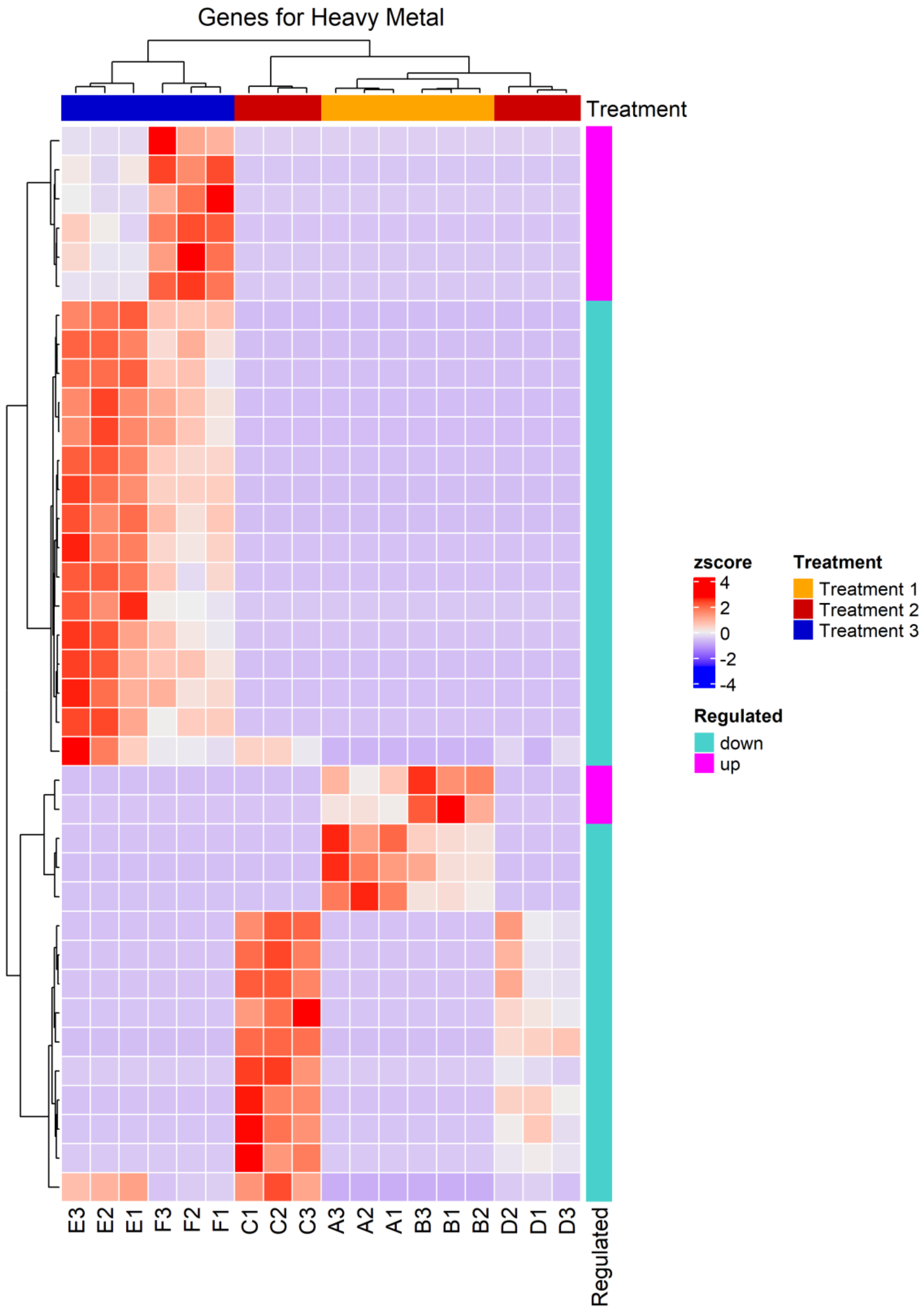

Supplementary Figure S3. Heatmap for Metal ion transport-related genes.

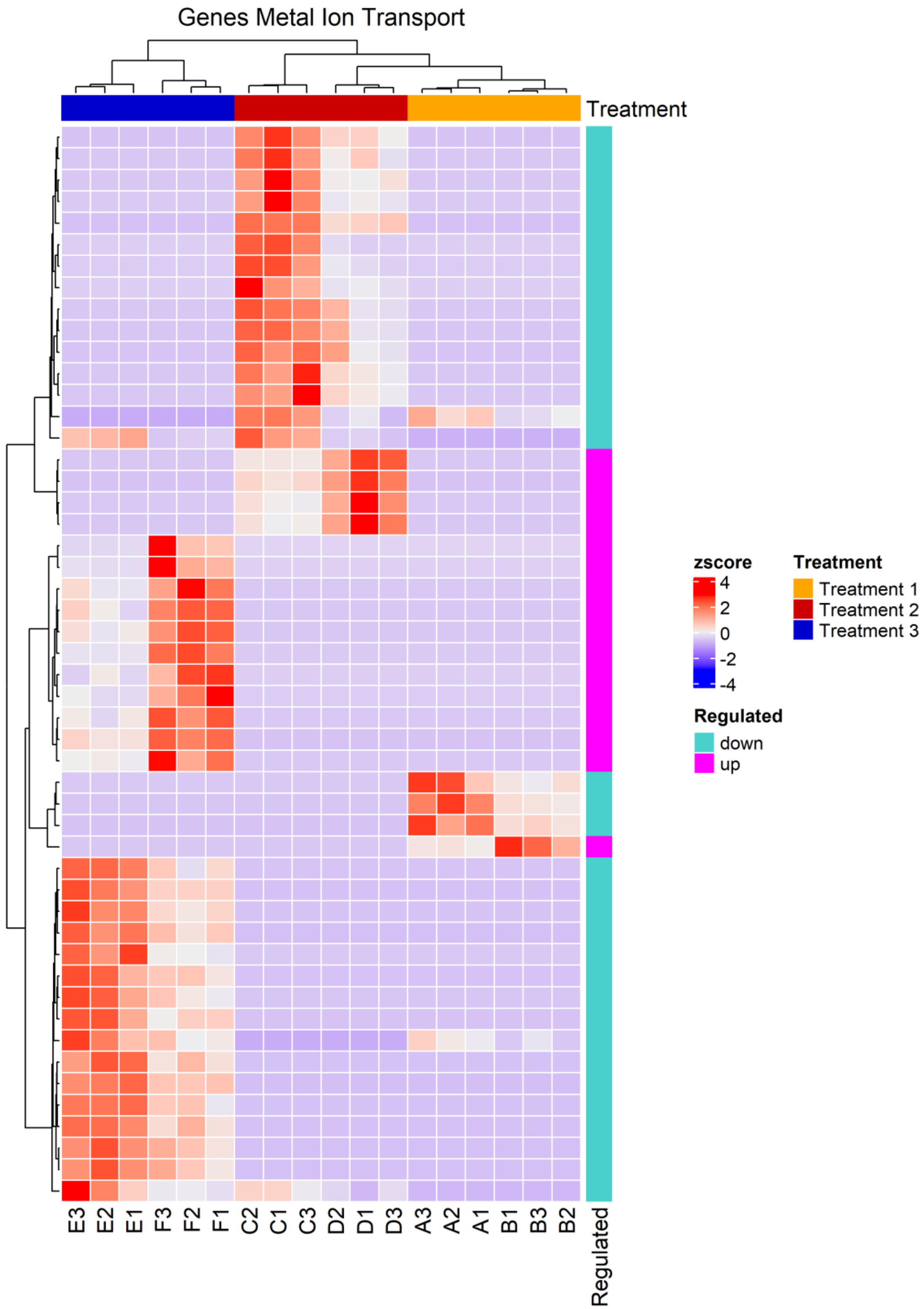

Supplementary Figure S4. Heatmap for Metal ion Auxin related genes.

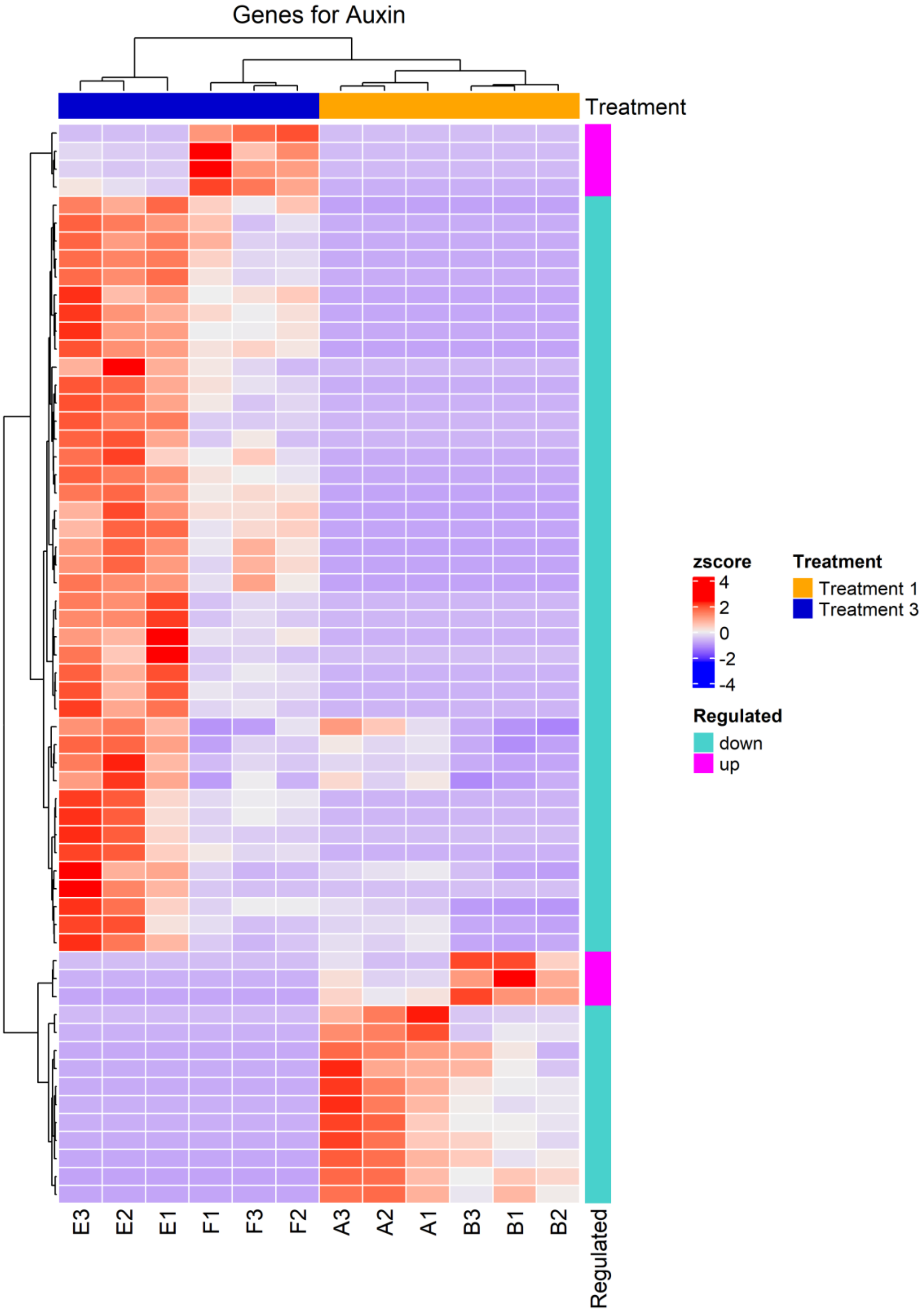

Supplementary Figure S5. Heatmap for ABC transporters pathways genes.

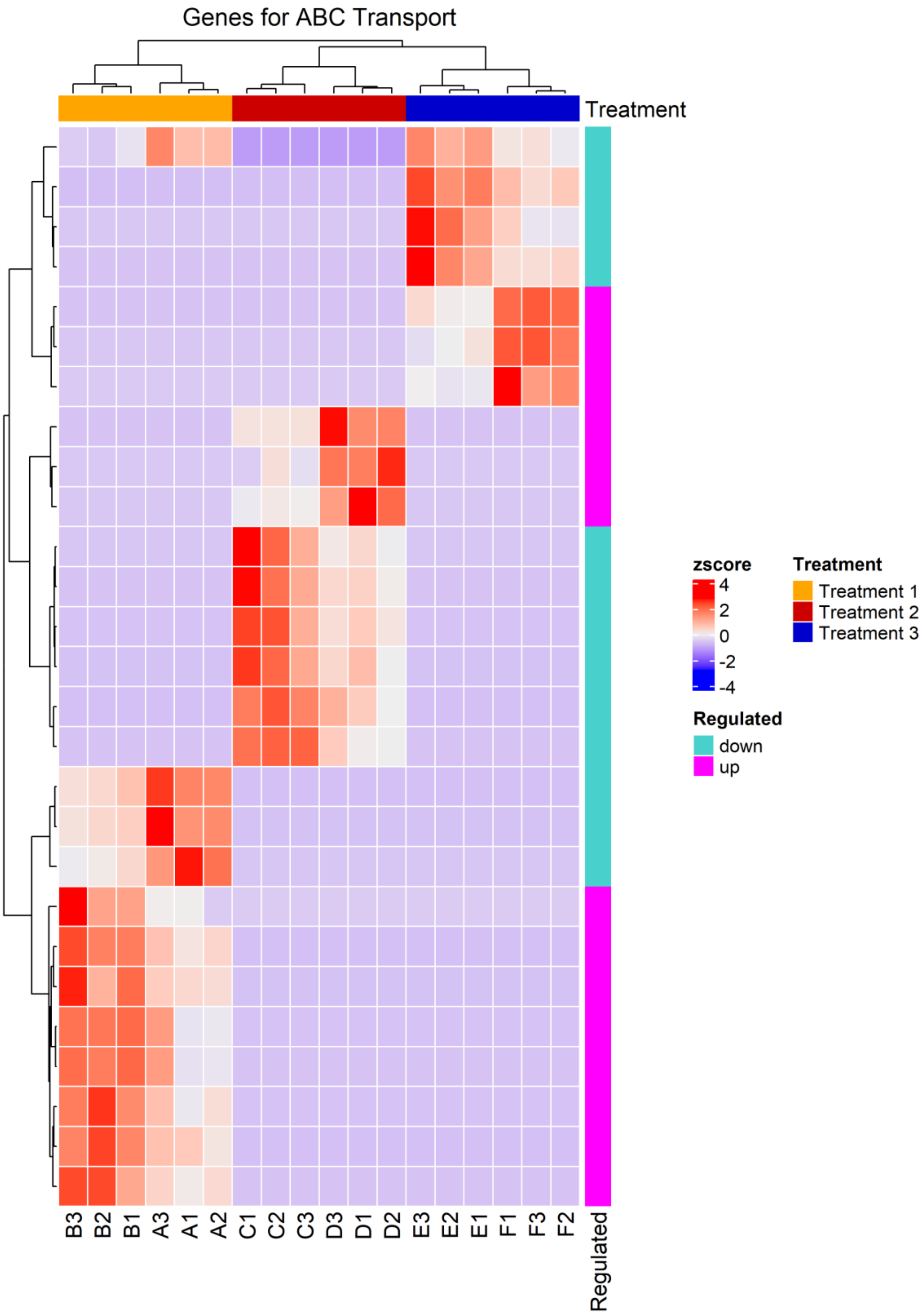

Supplementary Figure S6. Heatmap for Peroxidase activity-related genes.

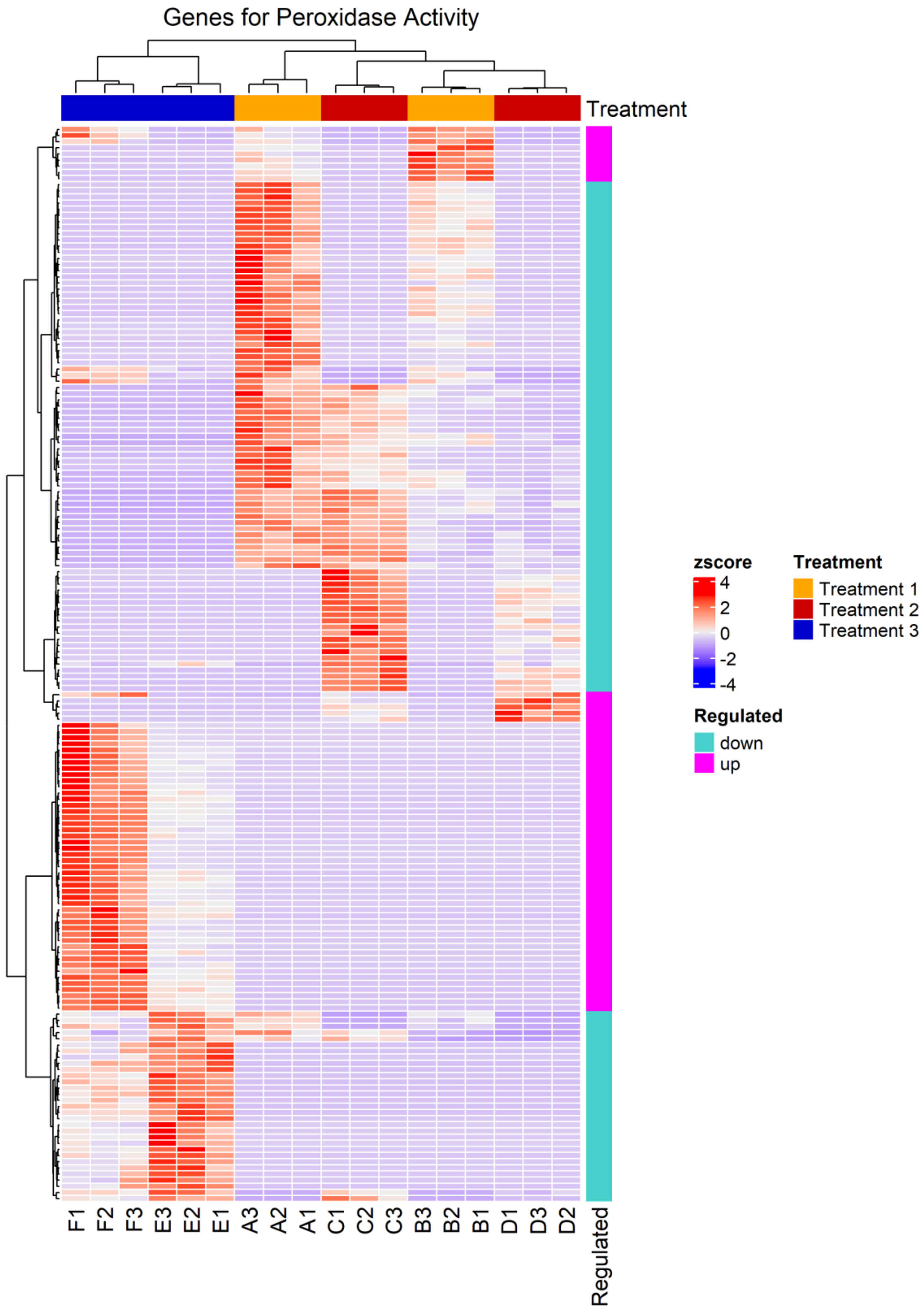

Supplement: Supplementary file 1 [file plants-12-00642-s001.zip › plants-2079076-supplementary.pdf]
